# Evaluation of Bone Density for Primary Implant Stability Using a Newly Designed Drill: An In Vitro Study on Polyurethane Bone Blocks

**DOI:** 10.1002/cre2.70048

**Published:** 2024-11-28

**Authors:** Kaien Wakamatsu, Kazuya Doi, Reiko Kobatake, Yoshifumi Oki, Kazuhiro Tsuga

**Affiliations:** ^1^ Department of Advanced Prosthodontics Hiroshima University Graduate School of Biomedical and Health Sciences Hiroshima Japan

**Keywords:** Bone density, Dental implantation, Dental implants

## Abstract

**Objectives:**

Bone density is an important factor for long‐term implant success. Peri‐implant bone density evaluation before implant placement can be useful for treatment planning, such as the selection of proper implant size or drilling protocol in each case. In this study, we aimed to establish an objective intraoperative bone density evaluation method by measuring the drilling torque value using a newly designed density measurement drill.

**Materials and Methods:**

Drilling torque value measurement was performed intraoperatively using three types of drills; two previously reported drills and a newly designed drill as a density measurement drill. Polyurethane bone blocks of different densities (D1–D4) were used in this experiment. After the measurement, implants were inserted based on the scheduled plan, and insertion torque (IT) and implant stability quotient (ISQ) were measured to assess primary implant stability.

**Results:**

The drilling torque value increased with the bone blocks' density, and there were significant differences among different densities in all groups (*p* < 0.05). The drilling torque value showed a positive correlation with IT in all groups (*p* < 0.05). In addition, the drilling torque value increased with the increase in ISQ in all groups.

**Conclusions:**

Within the limitations of this study, a newly designed density measurement drill was able to classify D1–D4 in polyurethane bone blocks despite its narrow diameter, and an objective intraoperative bone evaluation can be achieved. An intraoperative assessment of the drilling torque value can predict primary implant stability and provide valuable information for intraoperative treatment planning, such as undersized drilling protocol and implant size change.

**List of Abbreviations**
ISQ: implant stability quotient
IT: insertion torque
RFA: resonance frequency analysis
SD: standard deviations

## 1 Introduction

Primary implant stability plays an essential role in successful osseointegration and determination of long‐term implant success (Friberg, Jemt, and Lekholm 1991; Javed et al. 2013). Primary implant stability is influenced by bone quantity, bone density and quality, implant design, and surgical protocols (Roos, Sennerby, and Albrektsson 1997; Meredith 1998; Sennerby and Roos 1998). Among these, bone quantity and bone density have the highest influence on primary implant stability. A favorable primary implant stability cannot be obtained if the bone density has reduced because of osteoporosis (Oue et al. 2015). On the other hand, in dense bone, primary implant stability can be obtained; however, there can be a problem because of frictional overheating during surgical procedures (Eriksson and Albrektsson 1983). Furthermore, less vascularized bone is unfavorable for the achievement of osseointegration; therefore, evaluation of bone density is important for implant treatment planning. Preoperatively, panoramic radiography and computed tomography (CT) are performed to obtain information on bone characteristics and anatomical structures, and can be used for preoperative implant planning (Hatcher, Dial, and Mayorga 2003; Shahlaie et al. 2003; Turkyilmaz, Tözüm, and Tumer 2007; de Oliveira et al. 2008). However, these methods, which indirectly evaluate the implant placement site, may not accurately reflect the condition of the peri‐implant bone structures. Intraoperatively, bone characteristics are evaluated based on the tactile sensation by the surgical operator when preparing the implant socket. The evaluation based on the bone‐cutting sensation is subjective and has the disadvantage that it cannot be quantified. In recent years, there have been some reports of attempts to evaluate bone density by measuring the torque values using a cutting drill. Previous reports have described the intraoperative evaluation of bone density using a measuring drill (Iezzi et al. 2015; Di Stefano and Arosio 2016; Orlando et al. 2019; Di Stefano et al. 2021). A correlation exists between the measured torque value and bone density, and it could be expected to be useful for bone density evaluation. In our previous study using simulated bone with different densities, the torque value measured by the commercial tap drill correlated with density and primary implant stability, indicating that the intraoperative evaluation using a tap drill may be useful to evaluate primary implant stability (Wakamatsu et al. 2022). These studies showed that the bone density could be classified by torque value measurement during drilling; however, the intraoperative evaluations were performed using 3.0 mm diameter drills. These drills may limit the choice of drilling protocol to enhance the primary implant stability in cases with low bone density. Normally, an undersized drilling technique is used in cases of low bone density (Tabassum et al. 2010; Stocchero et al. 2016; Huang et al. 2020). The protocols of implant manufacturers recommend undersized drilling; for example, the implant site is prepared to 2.8 mm diameter before placement of a regular size implant for low bone density (Alghamdi, Anand, and Anil 2011). For this reason, a narrow diameter of the measurement drill is required to make treatment decisions based on the results of intraoperative bone density evaluation. However, a narrow‐diameter drill results in a smaller detected torque value, making it more difficult to classify bone density. Therefore, we developed a newly designed drill with a narrow diameter and an effective drill shape for torque detection (density measurement drill) and investigated the density evaluation. The information about peri‐implant bone density before implant placement can be useful for treatment planning, such as the selection of proper implant size or drilling protocol in each case.

The purpose of this study was to establish an objective intraoperative bone density evaluation method by measuring the drilling torque value using a newly designed density measurement drill.

## 2 Materials and Methods

### 2.1 Materials and Measurement Devices

Solid rigid polyurethane bone blocks of different densities according to Misch classification (D1: 0.48; D2: 0.32; D3: 0.16; and D4: 0.08 g/cc) (block size: 13 cm × 18 cm × 4 cm, blocks SAW 1522‐23/01/03/04 Sawbones; Pacific Research Laboratories, Inc., Washington, USA) were prepared as artificial substitutes. This material exhibits mechanical properties similar to those of human cancellous bone, as described in the ASTM F1839‐08 standard specification.

**Figure 1:**
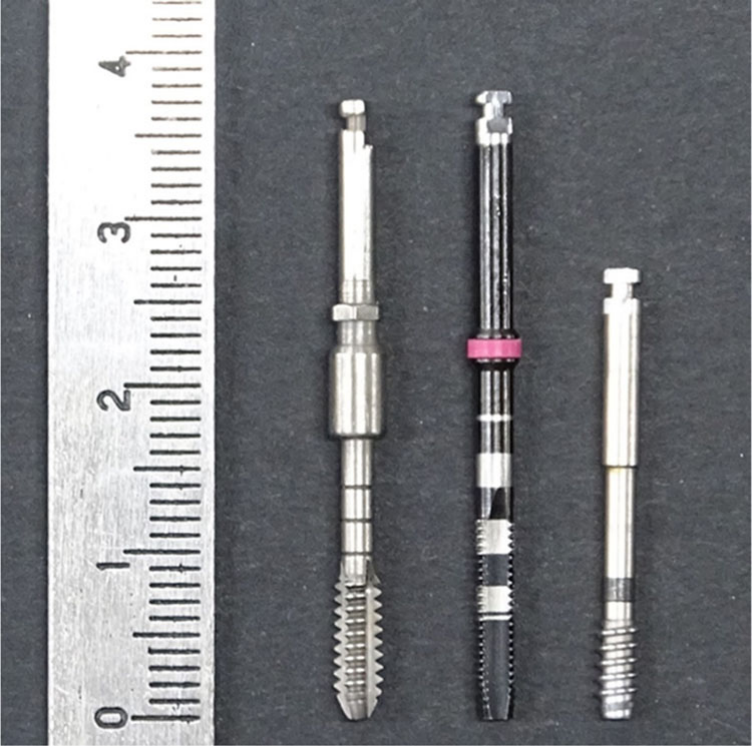
Measurement drills. The left drill: φ3.0 mm reading drill, which has grooves in the blade and the tip is tapered (Control 1 group). The center drill: φ3.0 mm tap drill, which has grooves in the blade and the tip is slightly tapered (Control 2 group). The right drill: φ2.7 mm density measurement drill, which has a spiral structure blade and cylindrical shape with a flat tip (Test group).

The drilling torque value was measured using three types of drills two previously reported drills and a newly designed density measurement drill (Figure 1). As the Control 1 group, φ3.0 mm reading drill (IDI evolution, Milano, Italy) dedicated to torque measurement was used. The reading drill was reported by Iezzi et al. as a drill used specifically for bone density evaluation (Iezzi et al. 2015). As a Control 2 group, φ3.0 mm tap drill (GC Corp., Tokyo, Japan) was used, which was applied to bone density evaluation in our previous study (Wakamatsu et al. 2022). As a test group, a φ2.7 mm density measurement drill (original manufactured; Nakanishi Inc., Tochigi, Japan) was prepared to evaluate bone density accurately with a narrow diameter drill. The drilling torque value was recorded by a computerized surgical implant motor device (Surgic Pro2; Nakanishi Inc.). This device could detect a low torque of 1.0 N cm or less. A pure titanium implant with a machined surface (Brånemark System 3.75 × 10 mm RP; Nobel Biocare, Kloten, Switzerland) was used in this study.

### 2.2 Measurement of the Drilling Torque Value and Implant Placement

**Figure 2:**
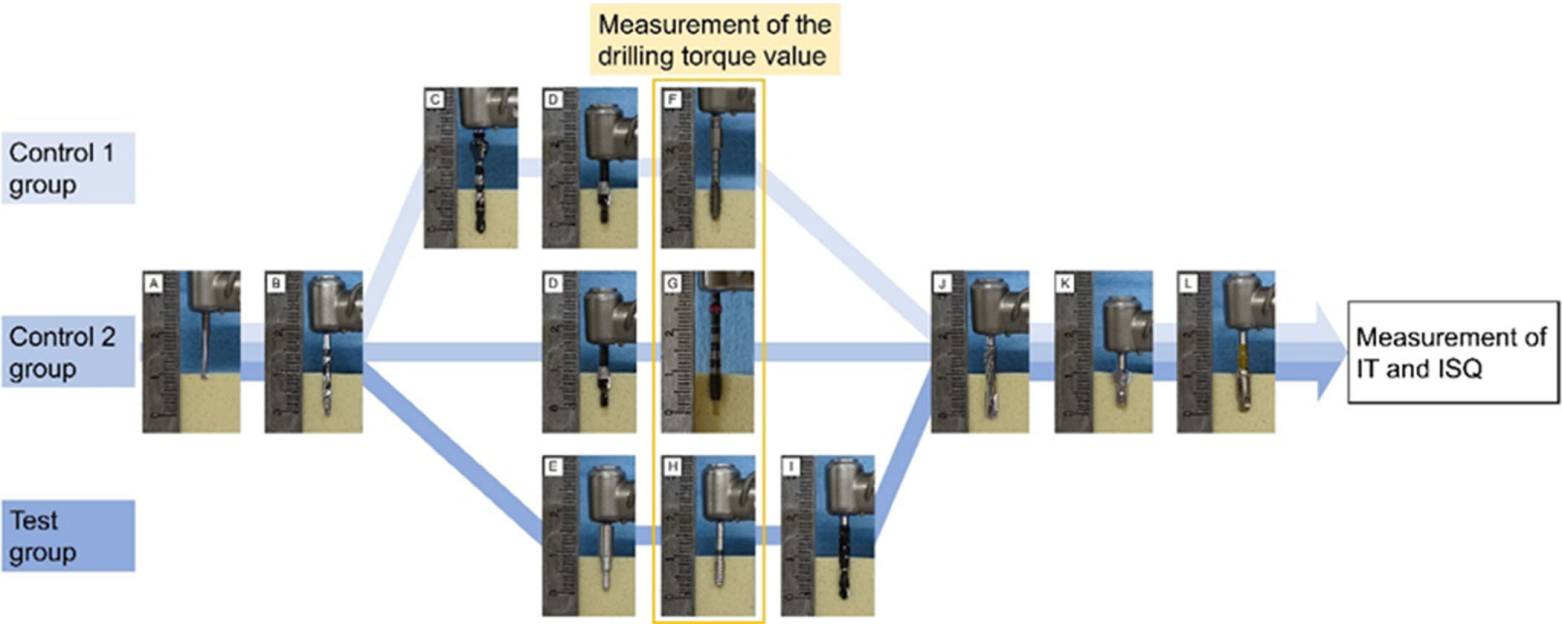
Drilling procedures for each measurement drill. Figures show the cross section of the center part of the drilling positions. Drilling procedures for the Control 1 group is “ABCDFJKL,” Control 2 group is “ABDGJKL,” and Test group is “ABEHIJKL.” (A) Round‐shaped drill (φ2.0 mm) was used to determine the drilling site. (B) Twist drill (φ2.0 mm) was used for preparing the implant socket up to a depth of 10 mm. (C) Twist drill (φ2.3 mm) was used for preparing the implant socket according to the torque measurement protocol of φ3.0 mm reading drill. (D) Pilot drill (φ3.1 mm) was used to drill up to 4 mm to remove the upper part of the bone block corresponding to the cortical bone area completely. (E) Pilot drill (φ2.8 mm) was used to drill up to 4 mm to remove the upper part of the bone block corresponding to the cortical bone area completely. (F) Reading drill (φ3.0 mm) was used to measure the drilling torque value. (G) Tap drill (φ3.0 mm) was used to measure the drilling torque value. (H) Density measurement drill (φ2.7 mm) was used to measure the drilling torque value. (I) Twist drill (φ2.4/2.8 mm) was used for preparing the implant socket. (J) Twist drill (φ3.0 mm) was used for preparing the implant socket. (K) Counterbore drill was used to drilling the upper part of the bone block. (L) Implant placement.

Drilling procedures for each measurement drill are shown in Figure 2. Measurement of the drilling torque value and implant placement were performed. To prevent the influence of structural changes on bone blocks, the implant sockets were placed at a distance of more than 15 mm from each other.

### 2.3 Control 1 Group

The reading drill, which has been reported as a specific drill for bone density evaluation, was used in the Control 1 group. The drill has grooves in the blade with a tapered tip, and its structure is similar to that of a tap drill. First, φ2.0 mm round‐shaped drill (Nobel Biocare) was used to determine the drilling site. Subsequently, φ2.0 mm (original manufactured) and φ2.3 mm twist drill (IDI evolution) were used for preparing the implant socket according to the torque measurement protocol of the φ3.0 mm reading drill. The depth was set at 10 mm to avoid picking up resistance torque when hitting the bottom of the measurement drill. Subsequently, φ3.1 mm pilot drill (GC Corp.) was used to drill up to 4 mm to remove the upper part of the bone block corresponding to the cortical bone area completely. The diameter of the pilot drill was larger than that of the reading drill; therefore, there was no effect of the upper 4 mm area when measuring the torque. After preparation, the drilling torque value was measured using a φ3.0 mm reading drill. The drilling torque value was measured at a speed of 35 rpm under continuous water irrigation until 7 mm (measurement depth: 3 mm), and the peak torque value was recorded.

### 2.4 Control 2 Group

The drilling preparation was performed before drilling torque value measurement according to the protocol of φ3.0 mm tap drill in our previous study, in the order of φ2.0 mm round drill, φ2.0 mm twist drill, and φ3.1 mm pilot drill. After preparation, the drilling torque value was measured using φ3.0 mm tap drill, in the same way.

### 2.5 Test Group

**Figure 3:**
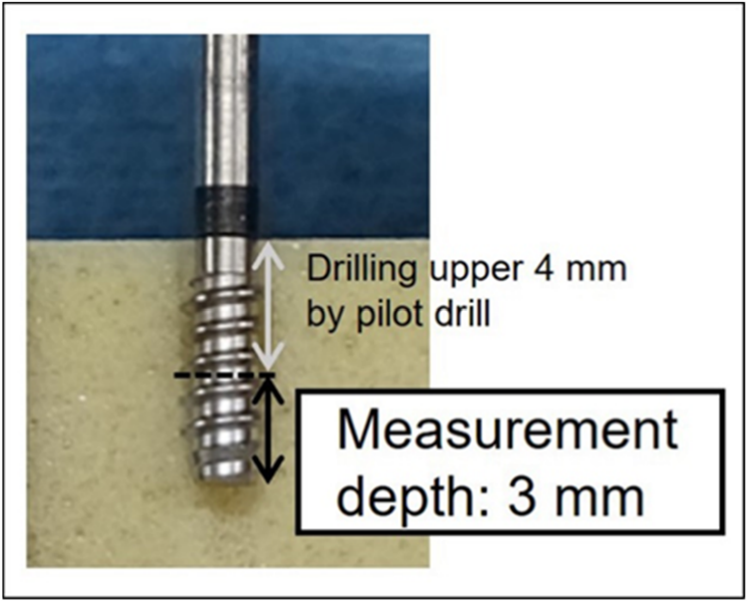
Cross‐section of measurement depth in the test group. Measurement was conducted under the same conditions in the control groups. The upper part (4 mm) was drilled using a pilot drill, and the lower part (3 mm) was included in the measurement depth.

The drilling preparation was performed using φ2.0 mm round drill and φ2.0 mm twist drill. Subsequently, a φ2.8 mm pilot drill (original manufactured) was used to remove the upper 4 mm area of the bone block. The pilot drill was made with a diameter suitable for the density measurement drill, and the stopper made it possible to strictly define the conditions of the measurement depth. After preparation, the drilling torque value was measured using a φ2.7 mm density measurement drill, in the same way. The cross‐section of measurement depth is shown in Figure 3.

### 2.6 Evaluation of Primary Implant Stability

After the drilling torque value was measured, drilling for implant placement was performed according to the instructions for the Brånemark System implants in medium‐density bone. In the test group, the 2.4/2.8 mm twist drill (Nobel Biocare) was used. Subsequently, a 3.0 mm twist drill (Nobel Biocare) and counterbore drill (Nobel Biocare) were used before implant placement in all groups. All drilling formation steps were performed under continuous water irrigation at a speed of 1200 rpm. The implant placement was performed at a speed of 35 rpm, and a peak insertion torque (IT) was recorded during implant insertion. After implant placement, the implant stability quotient (ISQ) was measured via resonance frequency analysis (RFA) using an Osstell device (Osstell AB, Gothenburg, Sweden), according to the manufacturer's instructions. In this study, a single drill was used for all the measurements, and bone blocks were used in a random order. Each measurement was performed five times by four experienced operators (*n* = 20).

### 2.7 Statistical Analysis

Comparison of the drilling torque values in each group and each different density were evaluated using one‐way analysis of variance (ANOVA), followed by Tukey's post‐hoc test. Pearson's correlation and linear regression analyses were performed to investigate the relationship between the drilling torque value and primary implant stability. The calculations were performed using Prism v7 (GraphPad, La Jolla, CA). All data were expressed as mean (standard deviation, SD) values, and statistical significance was set at *p* < 0.05.

## 3 Results

### 3.1 Density Classification

The classification of D1–D4 bone blocks' density using each measurement drill is shown in Table 1. The drilling torque value in the Control 1 group was as follows: D1 = 6.06 (0.47) N cm, D2 = 2.50 (0.19) N cm, D3 = 0.96 (0.24) N cm, and D4 = 0.69 (0.14) N cm. The drilling torque value in the Control 2 group was as follows: D1 = 7.47 (0.82) N cm, D2 = 2.92 (0.37) N cm, D3 = 1.23 (0.27) N cm, and D4 = 0.67 (0.19) N cm. The drilling torque value in the Test group was as follows: D1 = 9.18 (0.82) N cm, D2 = 3.27 (0.41) N cm, D3 = 1.19 (0.24) N cm, and D4 = 0.76 (0.19) N cm. The drilling torque value increased with the bone blocks' density, and there were significant differences in D1–D4 in all groups (*p* < 0.05). The density measurement drill was able to differentiate the bone blocks' density including low density despite its narrow diameter.

**Table 1:** Density classification by drilling torque.

	D1	D2	D3	D4
Control 1 group	6.06 (0.47)	2.50 (0.19)	0.96 (0.24)	0.69 (0.14)
Control 2 group	7.47 (0.82)	2.91 (0.37)	1.23 (0.27)	0.67 (0.19)
Test group	9.18 (0.82)	3.27 (0.41)	1.19 (0.24)	0.76 (0.19)

*Note:*Drilling torque value (N cm), Mean (SD). *p* < 0.05 was considered statistically significant. Control 1 group: Statistically significant between each. Control 2 group: Statistically significant between each. Test group: Statistically significant between each.

### 3.2 Drilling Torque Value in D1–D4

**Figure 4:**
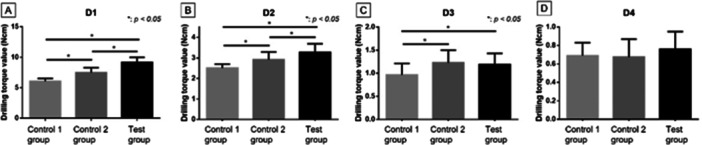
Drilling torque value at each block. (A, B) Significant differences were observed among all groups in D1 and D2 (*p* < 0.05). (C) The Test group and the Control 2 group showed higher values in D3 (*p* < 0.05). (D) There was no significant difference among all groups in D4.

A comparison of the drilling torque value with respect to each density is shown in Figure 4. The drilling torque values of the Test group in D1 and D2 were larger than those obtained with the control groups (*p* < 0.05). On the other hand, the Test group and the Control 2 group showed higher values in D3 (*p* < 0.05), and there was no significant difference among the three groups in D4.

### 3.3 Correlation Between Drilling Torque Value and Primary Implant Stability

**Figure 5:**
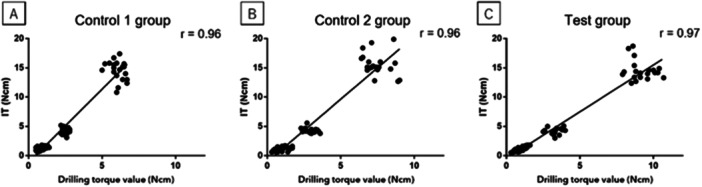
Drilling torque value and IT. A positive correlation was observed between drilling torque value and IT in all groups (*p* < 0.05).

**Figure 6:**
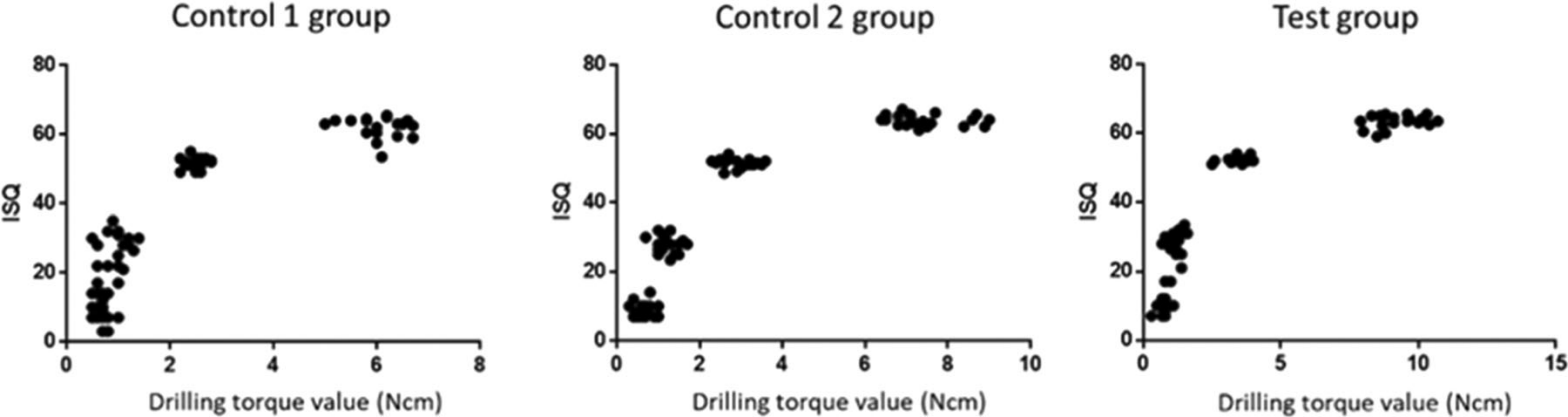
Drilling torque value and ISQ. The higher drilling torque value, the higher ISQ.

A correlation between drilling torque value and primary implant stability is shown in Figure 5. The drilling torque value showed a positive correlation with IT in all groups, and the correlation coefficients were *r* = 0.96, 0.96, and 0.97, respectively (*p* < 0.05, Figure 5A–C). In addition, the drilling torque value increased with an increase in ISQ in all groups (Figure 6).

## 4 Discussion

In this study, solid rigid polyurethane bone blocks of different densities were used. These bone blocks have been used in several previous reports and are classified according to their density as D1: 0.48 g/cc, D2: 0.32 g/cc, D3: 0.16 g/cc, and D4: 0.08 g/cc (Möhlhenrich et al. 2015, 2016; Comuzzi et al. 2019). We fabricated a newly designed density measurement drill with an effective drill shape for torque detection. The density measurement drill had a diameter of 2.7 mm, which was smaller than previously reported drill diameters. As a control, we used the reading drill and tap drill. These drills have been previously reported for use in bone density evaluation, and both have a diameter of 3.0 mm. Evaluation using these drills may limit subsequent treatment protocol due to their diameter. In this study, it was possible to classify the density using the φ2.7 mm density measurement drill. The drilling torques using the φ2.7 mm density measurement drill showed higher values in D1 and D2 compared with those of the control drills and showed the same level as those of the control drills in the low density of D4. This may be because the φ2.7 mm density measurement drill had no vertical groove structure resulting in higher frictional resistance, and it could detect the torque value comparable to that of the control drills despite its narrow diameter. Previous reports have evaluated bone density using φ3.0 mm drills; however, there are no reports of evaluation using the φ2.7 mm drill. On the other hand, in cases of low bone density such as D4, implant manufacturers recommend an undersized drilling protocol; for example, the implant site is prepared to 2.8 mm diameter before placement of a regular size implant (Alghamdi, Anand, and Anil 2011). Therefore, evaluation using the φ3.0 mm drill is difficult to apply, and a narrow measurement drill is desirable for selecting the drilling protocols based on the measurement results. Evaluation using the φ2.7 mm drill is compatible with the undersized drilling protocol and has the advantage of not interfering with subsequent drilling protocols before implant placement.

In clinical practice, the decision whether to change the drilling protocol and implant size intraoperatively is determined based on subjective evaluation during drilling. Previous studies regarding subjective evaluation during drilling of the implant site have reported that classification of low bone density is difficult. Trisi et al. examined bone density for subjective assessment and histomorphometric analysis and reported that D1 and D4 could be recognized easily; however, D2 and D3 were difficult to detect (Rao and Rao 1999). Alsaadi et al. examined the tactile sensation during high‐speed drilling and implant stability, and reported the recognition of D1 and D4; however, low bone density, such as D3 and D4, was difficult to recognize (Alsaadi et al. 2007). From the results of this experiment, an intraoperative objective evaluation can be achieved based on the density classification using the φ2.7 mm density measurement drill.

In cases of dense bone condition, high torque values may damage the bone tissue. Very high torque values in implant placement may compress the surrounding bone and prevent subsequent osseointegration (Cha et al. 2015; Ikar et al. 2020). To prevent excessive torque, a tap drill that is equivalent to the size of the implant should be used to cut the threads before implant placement in cases of dense bone condition. In this bone density evaluation, the torque value was around 10 N cm at D1, which was not a high torque. However, when high density is expected, a torque limiter should be used to prevent very high torque.

The primary implant stability was evaluated via IT and ISQ. IT is a combination of the resistance from the cutting edges of the apex and friction from the implant body, and the measurement of IT can be used to evaluate the bone quality and primary implant stability (O'Sullivan, Sennerby, and Meredith 2000). RFA is a noninvasive quantitative method, and the RFA values are calculated as ISQ and expressed as a number between 1 and 100. We reported that the drilling torque value using the tap drill correlated with subsequent primary implant stability (Wakamatsu et al. 2022). Similarly, in this study, the drilling torque value of the density measurement drill correlated with the primary implant stability. Therefore, an intraoperative evaluation of the drilling torque value can predict primary implant stability and provide valuable information for intraoperative treatment planning, such as undersized drilling protocol and implant size change. Furthermore, some surgical techniques have been reported in the case of insufficient bone volume and low bone density. Split crest procedure is used in cases of insufficient bone width, and the combined use of ultrasonic bone surgery has been reported to have well bone‐cutting efficiency, especially in soft type bone, and high success rates of implant (Blus et al. 2010). Also, osseodensification technique has been reported as a new drilling method to increase bone volume and density, and its utility has been demonstrated (Fontes Pereira et al. 2024). Intraoperative objective density evaluation of this study may be useful for these techniques.

## 5 Conclusion

Within the limitations of this study, a newly designed density measurement drill was able to classify D1–D4 in polyurethane bone blocks despite its narrow diameter, and an objective intraoperative bone evaluation can be achieved. Furthermore, the drilling torque value correlated with primary implant stability, indicating that the density measurement drill may be useful in considering implant treatment planning.

## Author Contributions

Kazuya Doi contributed to the study concept and design. Kaien Wakamatsu, Kazuya Doi, Reiko Kobatake, and Yoshifumi Oki participated in the data collection. Kaien Wakamatsu and Kazuya Doi checked the data. Reiko Kobatake performed the statistical analyses. Kaien Wakamatsu, Kazuya Doi, and Reiko Kobatake drafted the manuscript. Kaien Wakamatsu, Kazuya Doi, Reiko Kobatake, Yoshifumi Oki, and Kazuhiro Tsuga reviewed the findings of data analyses, participated in writing the manuscript, and approved the final draft of the manuscript submitted to the journal.

## Consent

The authors have nothing to report.

## Conflicts of Interest

The authors declare no conflicts of interest.

## Data Availability

Data sharing is not applicable to this article as no data sets were generated or analyzed during the current study.
